# New Treatments for Myelodysplastic Syndromes

**DOI:** 10.4084/MJHID.2010.021

**Published:** 2010-08-11

**Authors:** Francesco D’Alò, Mariangela Greco, Marianna Criscuolo, Maria Teresa Voso

**Affiliations:** Istituto di Ematologia, Università Cattolica Sacro Cuore, Rome, Italy

## Abstract

In the last decade, significant advances have been made in the treatment of patients with Myelodysplastic Syndromes (MDS). Although best supportive care continues to have an important role in the management of MDS, to date the therapeutic approach is diversified according to the IPSS risk group, karyotype, patient’s age, comorbidities, and compliance. Hematopoietic growth factors play a major role in lower risk MDS patients, and include high dose erithropoiesis stimulating agents and thrombopoietic receptor agonists. Standard supportive care should also include iron chelating therapy to reduce organ damage related to iron overload in transfusion-dependent patients. Biologic therapies have been introduced in MDS, as lenalidomide, which has been shown to induce transfusion independence in most lower risk MDS patients with del5q. Hypomethylating agents have shown efficacy in INT-2/high risk MDS patients, reducing the risk of leukemic transformation and increasing survival. Other agents under development for the treatment of MDS include histone deacetylase inhibitors, farnesyltransferase inhibitors, clofarabine and ezatiostat.

## Introducion:

Myelodysplastic Syndromes (MDS) are a heterogeneous group of clonal disorder of hematopoiesis characterized by ineffective hematopoiesis, morphological signs of dysplasia, peripheral cytopenia and increased risk of develop Acute Myeloid Leukemia (AML). MDS is a typical disorder of the elderly and most patients are over 70 years of age at diagnosis. Up to recently, MDS treatment was mainly supportive, including transfusion of blood components and antibiotics, while a minority of younger patients, with good performance status and without significant comorbidities, was eligible for more intensive treatment including chemotherapy and allogenic stem cell transplantation, but results were often dismal. In the last decade, the treatment of MDS has significantly improved by the introduction of new agents and by different use of old drugs, so that the pharmacological armamentarium, as well as the number of MDS patients suitable for treatment are definitely wider than in the past. This has been made possible by new insights in the biology and pathogenesis of MDS, although many aspects of the efficacy of the new drugs are still unknown and under investigation.^1^

Considering the heterogeneity of nosographic entities representing MDS, risk stratification is essential to target the treatment according to the risk of AML evolution and blood transfusion requirement. In the last decade, the risk evaluation score IPSS has guided physicians on therapeutic decisions, stratifying MDS patients into Low, Intermediate-1 (INT-1), Intermediate-2 (INT-2) and High risk, according to number of peripheral blood cytopenias, bone marrow blast percentage and karyotype.^2^ More recently, the WHO classification-based prognostic scoring system (WPSS) has included the WHO morphologic classification and underlined the importance of transfusion requirements as prognostic factor for survival in MDS patients.^3^

Besides disease-related factors, therapeutic decisions in MDS patients should also take into account other patients’ related factors, as age, performance status, comorbidities and quality of life. Patients’ compliance to the treatment is definitively pivotal to achieve therapeutic results. According to the IPSS, current guidelines have been developed for MDS treatment.^4^,^5^

## Erithropoiesis stimulating agents (ESAs):

MDS patients with low-INT1 IPSS risk, hemoglobin levels lower than 10 g/dl, and serum erythropoietin levels <500 mIU/ml should be treated according to current guidelines with high dose ESAs, as fixed weekly subcutaneous doses of 60–80,000U α- or β-erythropoietin or 300 μg darbepoetin.^4^,^5^

Three retrospective studies suggest a survival advantage for ESAs, possibly through their effect on minimizing transfusion needs and reducing iron overload. Jädersten at al.^6^ compared 121 Nordic patients with MDS treated with erythropoietin plus granulocyte colony-stimulating factor (G-CSF) to a disease and time-matched cohort of 237 patients from Pavia, Italy, who received no therapy. The erythroid response rate in the treated group was 39%, and the median response duration 23 months. Those receiving ESA-based therapy had a significant survival (OS) advantage (HR=0.61, 95% CI:0.44–0.83, *p*=0.002) without any difference in the risk of leukemic transformation. The Groupe Francophone des Myelodysplasies compared in a matched-pair analysis 200 ESA-treated patients with 200 patients from the ESA-untreated subjects used to define the IPSS, and again demonstrated improved OS in the ESA cohort (HR=0.43, 95% CI:0.25–0.72), despite no difference in rates of acute myeloid leukemia (AML) transformation.^7^ The Cleveland group performed a pooled analysis of 162 published trials enrolling lower-risk MDS patients over a 20-year period and compared 1587 patients treated with ESA-based therapy with 1005 patients treated with non-growth factor (NGF) approaches.^8^ Growth factors (GF) produced higher overall response rates (39.5% vs. 31.4% for NGF, *p*=0.019), while NGF yielded better CR/PR rates (25.6% vs. 9.1% for GF, p=0.03). Over 2 years follow-up, patients receiving ESA-based therapy demonstrated longer overall and progression-free survival.

Moreover, among transfusion-dependent MDS patients, a significant survival advantage has been demonstrated in patients responding to ESAs as compared to those resistant or to subjects never treated with EPO (median 52 months vs. 31 months, p<0.0095).^9^

A meta-analysis of 30 studies from 1990 to 2006 evaluating a total of 1314 MDS patients treated with epoetin alfa (22 studies, 925 patients) or darbepoetin alfa (8 studies, 389 patients) found no significant difference in the pooled erythroid response rates between the two agents (57.6% and 59.4%, respectively, p=0.828).^10^ Similarly, no differences in erythroid response rates was found between EPO as a single agent or combined with G-CSF or granulocyte-macrophage-colony-stimulating factor (GM-CSF).^11^ Furthermore, EPO monotherapy at higher doses of 60000 to 80000 U weekly produced significantly higher erythroid response rates (64.5%) compared with the standard dose of 30000 to 40000 U weekly either as a single agent (49%; p<0.001) or in combination with G-CSF/GM-CSF (50.6% p=0.007).^11^

The combination of ESAs and G-CSF should be considered only in less heavily transfused patients (less than 2U per month), with serum erythropoietin levels <500 mIU/ml, unresponsive to ESAs alone.^4^,^5^,^12^

Finally, G-CSF is useful in neutropenic MDS patients with recurrent or resistant infections but should not be used for routine infection prophylaxis.^13^

## Thrombopoietin receptor agonist:

Thrombocytopenia occurs at the time of initial diagnosis in 30% of MDS patients and represents an independent adverse prognostic factor for survival.^14^,^15^ Platelet function may be abnormal in MDS, making the presence of moderate to severe thrombocytopenia of greater concern. Platelet transfusions are the only current treatment option, but new approaches are under investigation.

Romiplostim is a peptibody, increasing platelet production through the thrombopoietin (TPO) receptor (c-Mpl). Clinical studies showed that romiplostim (Nplate®, Amgen) increases platelet counts in healthy individuals and in patients with chronic immune thrombocytopenia. In a recent phase I/II, multicenter, open-label, sequential-cohort, dose-escalation study, romiplostin at the weekly doses of 300, 700, 1000, or 1500 μg increased platelet counts in 44 MDS patients with low or INT-1 IPSS and mean baseline platelet counts ≤ 50 x 10^9^/L, treated with supportive care. Durable platelet response according to the International Working Group 2000 criteria, was achieved in 19 patients (46%) with reduction of the incidence of bleeding events and platelet transfusions. Treatment-related serious adverse events were reported in five patients (11%), all of whom in the 1500 μg dose cohort, including two AML progressions.^16^ Further studies need to clarify the role of thrombopoietin receptor agonist in management of thrombocytopenic MDS patients.

## Lenalidomide:

Lenalidomide is a 4-amino-glutarimide, analogue of the older immunomodulatory drug thalidomide. Supposed mechanism of action involves modulation of cellular response to receptor/ligand activation signals, via suppression of pro-inflammatory and pro-apoptotic cytokine generation, prevention of angiogenesis, altered cell adhesion to bone marrow stroma, and direct cytotoxicity against dysplastic clones.^1^

Four phase II trials investigated the efficacy of lenalidomide as single agent in more than 400 patients, with low/INT-1 MDS, with symptomatic or transfusion-dependent anemia, refractory to ESAs. Patients with 5q deletion are peculiarly responsive to lenalidomide. Best dose and schedule are 10 mg/day for 21 days every 4 weeks. Dose reduction was required in patients with renal failure and altered drug metabolism, especially because responding patients may experience severe dose-related neutropenia and thrombocytopenia due to selective elimination of the 5q- positive clone.^4^

In the phase II registration study including 148 patients with the 5q- deletion, lenalidomide was administered at a dose of 10 mg/day for a 28-day cycle or 10 mg/day for 21 days of a 28-day cycle.^17^ Erythroid response occurred in 76% patients, including 67% transfusion independence and 45% complete cytogenetic responses. The response to lenalidomide was rapid (median time to response, 4.6 weeks; range, 1 to 49) and sustained; the median duration of transfusion independence had not been reached after a median follow-up of 104 weeks. The maximum hemoglobin concentration reached a median of 13.4 g per deciliter (range, 9.2 to 18.6), with a corresponding median rise of 5.4 g per deciliter (range, 1.1 to 11.4). Similar data were reported by the results of French compassionate program (ATU) of lenalidomide in low/INT-1 risk MDS with del 5q, where 62/95 pts (65%) achieved erythroid response, including 60 (63%) transfusion independence (TI).^18^

In a double-blind randomized phase III trial (MDS-004), involving patients with Low-or INT-1-Risk MDS with del5q, lenalidomide at the dose of 10 mg/day for 21 days of each 28-day cycle was associated with better RBC-transfusion independence and cytogenetic responses than the dose of 5 mg/day for 28 days of each 28-day cycle, with a comparable safety profile, and should be considered as a starting dose.^19^

Response rates are lower in low/INT-1 MDS patients without 5q deletion. In a phase II study involving 214 transfusion-dependent low/INT-1 MDS non-5q deleted patients, lenalidomide administered at 10 mg daily or 10 mg on days 1 to 21 of a 28-day cycle induced 43% overall response rate (ORR), including 26% transfusion independence and 17% reduction of more than 50% transfusion requirements.^20^

Response to lenalidomide have been described also in INT-2/high-risk MDS with 5q deletion. In a phase II trial, of 47 patients with higher-risk MDS who received lenalidomide 10 mg/day, 13 (27%) achieved hematologic response including 12 RBC-transfusion independence. Six of 9 patients with isolated del 5q achieved complete remission, versus 1 of 11 and none of 27 patients with one or more than one additional abnormality, respectively (*p*<0.001).^21^

The karyotype-selective therapeutic effect of lenalidomide toward MDS clone carrying del5q abnormality has been related to its inhibitory activity on two phosphatases, Cdc25C and PP2Acalpha, which are codified by the common deleted region (CDR) and work as coregulators of the G(2)-M checkpoint. Treatment of del(5q) AML cells with lenalidomide induced G(2) arrest and apoptosis, whereas there was no effect in nondel(5q) AML cells.^22^

Other major effects have been reported on RPS14, SPARC and other genes, including p53.

Sporadic MDS patients treated with lenalidomide progressed to AML. Although the rate of AML progression does not significantly differ from patients with similar characteristics, strict cytogenetic monitoring and the use of lenalidomide within controlled therapeutic programs (registries or clinical trials) are required.^4^

## Iron-chelation therapy:

Most patients with MDS receive RBC transfusions for symptomatic management of their anemia during the course of their disease. Transfusion requirement has been identified as a major negative prognostic factor in MDS patients and is associated with shorter survival and an increased risk for progression into AML.^3^ Moreover, chronic transfusion dependence leads to iron overload, which represents a serious treatment-related complication, causing clinical consequences as cirrhosis, dilated cardiomyopathy, and progressive dysfunction of the endocrine glands.^23^,^24^ This is of greatest concern in patients with lower-risk MDS whose expected survival is measured in years. Moreover, in patients undergoing myeloablative stem cell transplantation elevated pre-transplantation serum ferritin is strongly associated with lower overall and disease-free survival and increased transplant-related mortality.^25^

Case control studies, prospective surveys, and phase II studies indicate that iron chelation therapy reduces iron load as measured by changes in serum ferritin and may prolong overall survival.^26^,^27^

In a retrospective, single-institution analysis, chelation therapy emerged as the only significant factor improving overall survival in multivariate analysis in low- or INT-1-risk patients. Median survival was greater than 160 months in patients who received chelation therapy compared with 40 months in patients who did not (*p*<0.03).^26^

Similarly, in the prospective survey performed by the Groupe Français des Myélodysplasies (GFM), in 97 low /INT-1 regularly transfused patients, the median survival was 124 months for patients receiving chelation therapy vs 53 months for non chelated patients (*p*<0.0003). In the multivariate Cox analysis, adequate chelation was the strongest independent factor associated with improved OS.^27^

Chelation therapy significantly decreased serum ferritin levels over time in multicenter phase II clinical trials.^28^,^29^ In a phase II open-label, 3-year study, List et al.^28^ evaluated safety and efficacy of deferasirox (Exjade®, Novartis) 20 to 40 mg/kg per day in 176 RBC transfusion-dependent, low and INT-1 patients (US-03). Mean serum ferritin level significantly decreased from baseline (3397 ± 233 μg/mL) to month 12 (2501 ± 139 μ/mL). The labile plasma iron (LPI) concentration, a measure of the reactive species of NTBI, was reduced from 0.4 μmol/L at baseline to non-detectable at 3 months in all patients, and remained within the normal range for the remaining follow-up. The reduction of LPI and of reactive oxygen species is one of the mechanisms described for the improvement of erythropoiesis observed in some patients undergoing iron-chelating therapy.

The multicenter Exjade Evaluation of Patients’ Iron Chelation (EPIC) study recruited 1744 patients with transfusional hemosiderosis deriving from various types of anemia, including 341 MDS patients. Deferasirox was administered, based on transfusional iron intake, at 20 mg/kg/day for patients receiving 2–4 packed red blood cell units/month, while 10 or 30 mg/kg/day were recommended for patients receiving fewer or more transfusions, respectively. Dose adjustments were based on 3-month serum ferritin trends and continuous assessment of safety markers. Median serum ferritin after 1 year of deferasirox treatment significantly decreased by a median of 264 ng/mL from baseline (P<0.0001).^29^

Current NCCN guidelines recommend the use of deferasirox in the treatment of iron overload for low and INT-1-risk MDS patients, anticipated to receive, or having received greater than 20 RBC transfusions and/or who will reach serum ferritin level >2500 μg/L.^5^ The goal is to maintain ferritin < 1000 μg/L, thereby preventing accumulation of NTBI and the adverse effects on cardiac, hepatic, and endocrine function.

## Hypomethylating agents:

Azacitidine and decitabine were approved by the US Food and Drug Administration (FDA) for treatment of patients with MDS in 2004 and 2006, respectively. The European Medicines Agency (EMEA) formally approved azacitidine for MDS and AML treatment in March 2009. These agents are now used for MDS treatment in most countries in the developed world.^30^,^31^

The main biologic effect of these drugs consists in the inhibition of DNA methyltransferases followed by DNA hypomethylation and reactivation of the expression of epigenetically silenced tumor-suppressor genes. Furthermore, other mechanisms, such as direct cytotoxicity, DNA-damage response and apoptosis, are induced.^30^

Although hypomethylating agents have existed for about 40 years, their efficacy has been demonstrated in hematologic malignancies (especially MDS), just in the last 10 years (**Table 1**). The phase III, randomized, controlled trial, CALGB 9221 randomized 191 MDS patients in 2 arms, azacitidine 75 mg/m^2^ daily given subcutaneously for 7 days every 28 days versus best supportive care.^32^ Patients in the supportive care group were allowed to cross-over to azacitidine after 4 months, in case of disease progression. Responses occurred in 60% of patients on azacitidine (7% CR, 16% PR, 37% HI) and in 5% (HI) of those receiving supportive care (p < 0.0001). Median time to leukemic transformation or death was 21 months for azacitidine versus 12 months for supportive care (p=0.007). Transformation to AML occurred as the first event in 15% of patients on azacitidine and in 38% of patients receiving supportive care (p=0.001). Overall quality of life and social functioning, especially fatigue and psychological state, were significantly improved by azacitidine.^33^

Later on, data on 268 patients treated with azacitidine (75mg/m^2^/day for 7 days) in the CALGB 9221 and in 2 previous protocols (CALGB 8421, intravenous infusion and 8921, subcutaneous) were re-analyzed according to the IWG response criteria, for azacitidine marketing approval by the U.S. FDA.^34^ Global response (CR + PR) to azacitidine was 15.2%, rates were similar in all MDS subtypes and AML, and lasted at least 9 months. In addition, about 19% of azacitidine-treated patients achieved hematologic improvement (HI) and two thirds of transfusion-dependent patients became transfusion independent. Transformation into AML occurred in 44% of patients in the observation arm, versus 14% of patients randomized to azacitidine and 12% of those crossing-over to azacitidine after observation.

These data were confirmed by the AZA-001 phase III, international, multicentre, controlled, parallel-group, open-label trial, in 358 patients with higher-risk MDS. Patients were one-to-one randomly assigned to receive azacitidine (75 mg/m^2^ daily for 7 days every 28 days) or conventional care (best supportive care, low-dose cytarabine, or intensive chemotherapy).^35^ After a median follow-up of 21.1 months, median overall survival was 24.5 months for the azacitidine group versus 15.0 months for the conventional care group (hazard ratio 0.58; 95% CI 0.43–0.77; stratified log-rank p=0.0001). This study definitively showed that azacitidine increases overall survival in patients with higher-risk myelodysplastic syndromes, compared to conventional care. An oral preparation of azacitidine is in development.

The alternative hypomethylating agent decitabine, was used at 15 mg/m^2^ i.v. over 4 hours, three-times daily, for three consecutive days every 6 weeks, in the GMDSSG/EORTC 06011 randomized phase III trial. In this study, comparing low dose decitabine to supportive care in 233 patients over 60 years, with primary or secondary MDS or CMML, a 34% ORR (13% CR, 6% PR, and 15% HI) was obtained [36]. Probably due to the design of the study, allowing a maximum of 8 decitabine cycles, the decitabine dose and the absence of mainteinance treatment, no significant differences in survival and time to AML were observed. The D-0007 US registration trial using the same decitabine dose and schedule, showed similar response rates and a trend towards longer median time to AML progression or survival in patients treated with decitabine compared to supportive care alone.^37^ Higher responses rates were observed using decitabine i.v. at 20 mg/m^2^ daily for 5 consecutive days every 4 weeks in the outpatient setting, with 32% ORR and 18% HI.^38^,^39^

Treatment with hypomethylating agents (azacitidine/decitabine) was shown to be superior to intensive chemotherapy in terms of response rate and survival in patients with AML or high-risk MDS and chromosome 5 and 7 abnormalities.^40^

Current guidelines from the National Comprehensive Cancer Network (NCCN) (http://www.nccn.org) recommend azacitidine for at least 6 cycles, for patients with high-risk MDS, with decitabine as alternative.^5^ This recommendation is due to the survival advantage observed with azacitidine in the AZA-001 trial. Azacitidine and decitabine do not appear to be curative; continued therapy is necessary to maintain response.

Azacitidine was also shown to be a feasible and effective treatment for patients with lower risk MDS inducing 45–60% overall response rate according to different studies.^32^,^34^,^41^,^42^

## HDAC inhibitors:

Histones play an important structural role in the eukaryotic nucleus. Numerous histone posttranslational modifications, commonly known as the “histone code”, result in changes in the accessibility of associated DNA to transcription, and alter interactions of the nucleosome with chromatin-associated proteins. Among these modifications, deacetylation of lysine residues on histones has the potential to compact chromatin resulting in transcriptional gene repression.^30^

Histone Deacetylase (HDAC) inhibitors are a class of drugs which inhibit the activity of class I, class II and class IV HDACs. After HDAC inhibition with the resulting hyperacetylation of lysine residues in the histone tails, chromatin structure shifts to a transcriptionally active state, with re-expression of silenced genes. Additionally, it is likely that part of HDAC inhibitors’ effects on cellular profile might be independent of chromatin state modification and related to deacetylation of non-histone proteins. HDAC inhibitors may modify stability of the protein by altering chaperone protein function or preventing ubiquitinilation and proteosome degradation and may interfere with the subcellular localization, DNA-binding activity, protein-protein interaction of several non histone proteins, such as transcription factors or signal transducers, by increasing their acetylation. Finally HDAC inhibitors may induce growth arrest, differentiation or apoptosis in vitro and in vivo.^30^

Some compounds inhibit specific HDAC enzymes, such as entinostat (MS-275) or MGCD0103 (MethylGene), which selectively inhibit only class I HDACs. Other agents, such as panobinostat (LBH589), more broadly inhibit HDACs. In clinical trials, most HDAC inhibitors have been associated with gastrointestinal side effects, severe fatigue, thrombocytopenia and QTc prolongation as the most common treatment-related adverse events.^30^,^43^

Preliminary data in MDS and AML have bee reported for valproic acid, sodium phenylbutyrate, vorinostat (suberoylanilide hydroxamic acid, Zolinza) entinostat (SNDX-275/MS-275), belinostat (PXD101), romidepsin (FK228 / FR901229 / depsipepide), panobinostat (NVP-LBH589) and MGCD0103.^30^,^43^

HDAC inhibitors have shown limited single-agent activity in MDS, so that most expectations for these drugs derive from combination studies with hypomethylating agents or other agents with in vitro synergism.

The fatty acid sodium phenylbutyrate sodium as a continuous i.v. infusion was administered alone or in sequential combination with azacitidine to AML and MDS patients in small phase II studies, but because of the inconvenient schedule required to achieve biologically effective serum concentrations, sodium phenylbutyrate has not been further developed.^30^,^43^

Valproic acid has been used in monotherapy or in combination with ATRA or hypomethylating agents in MDS and AML patients. As single-agent, response were observed in 8 out of 18 patients, including 1 partial remission.^44^ In combination with ATRA, response rates were 6% in patients with Refractory Anemia with Excess Blast (RAEB) and and 16% in AML unfit to receive intensive chemotherapy.^45^ Hematologic improvement and stabilization of the disease was also observed. In the Italian multicenter GIMEMA trial MDS0205 combining azacitidine at standard doses with valproic acid in INT-2/high risk MDS patients, higher response rates were observed in patients reaching plasma concentration of 50 μg/mL of valproic acid on day 1 of azacitidine treatment, suggesting that the achievement of valproic acid therapeutic levels may increase azacitidine efficacy.^46^ A randomized study of 67 evaluable patients with MDS, AML, or CMML comparing decitabine at a dose of 20 mg/m^2^ daily for 5 consecutive days every 28 days with or without valproic acid showed no significant difference.^47^

Oral vorinostat (SAHA) at doses between 100 and 300 mg 2 or 3-times daily for 14 days followed by 1-week rest has been used in 41 patients with relapsed or refractory leukemias or MDS. Seven patients obtained hematologic improvement, including 2 CR and 2 CR with incomplete blood count recovery.^48^ Preliminary data on vorinostat combined with decitabine in AML patients showed only transient results in 30 patients with relapsed or refractory leukemia.^49^

Oral MGCD0103 has shown promising activity alone or in combination with azacitidine. Further development of this drug is uncertain due to significant cardiac side effects, including hemodynamically significant pericardial effusion, observed in the follow-up of a randomized study of azacitidine with or without MGCD0103.^43^

Entinostat, belinostat and romidepsin monotherapy have shown limited clinical activity in unselected AML/MDS patients in Phase I/II clinical trials.^43^ Panobinostat data in patients with MDS have not yet been reported, while 2 CR were reported in 62 AML patients in a Phase I/II study using oral panobinostat in advanced hematologic malignancies.^50^

## Other drugs:

Clofarabine is a halogenated purine analogue approved by FDA for the treatment of children with acute lymphoblastic leukemia in relapse. The proposed mechanism of action of clofarabine includes not just incorporation into DNA with induction of apoptosis, but also inhibition of ribonucleotide reductase and DNA synthesis.^43^ This drug has been explored in higher-risk MDS and AML. The MD Anderson group treated 32 MDS patients, 22 with INT-2/high risk disease, 20 patients with prior hypomethylating therapy failure, with three doses of clofarabine per os (40 mg/m^2^, 30 mg/m^2^ and 20 mg/m^2^ daily for 5 days every 4 to 8 weeks). ORR was 43%, including 25% CR, 9% HI and 9% clinical benefit. Response rates were lower in patients with a previous failure with hypomethylating agents. Common adverse events were gastrointestinal and hepatic disturbances and myelosuppression.^51^

Ezatiostat (TLK199, TER199, Telintra) is a glutathione analog exerting biologic effects by indirect activation of Jun-N-terminal kinase (JNK). Ezatiostat’s metabolite TLK117 binds to glutathione S-transferase P1-1 (GST P1-1) and causes its dissociation from JNK, activating JNK, with consequent induction of growth and maturation of hematopoietic progenitors. Ezatiostat has been recently tested in a phase I study including 45 lower-risk MDS patients. No dose-limiting toxicity was reached using 200 mg to 6000 mg daily per os for 7 days of a 21-day cycle, and common side effects were gastrointestinal. Seventeen patients (38%) achieved hematologic improvement using IWG criteria. Extended dosing schedules are being evaluated in a phase II study.^52^

Tipifarnib (R115777, Zarnestra) is a farnesyltransferase inhibitor currently in development for treatment of myeloid malignancies. The enzyme farnesyltransferase is important for membrane attachment of the Ras oncogene. Constitutive Ras activation and Ras hyperactivity contributes to cell proliferation and survival in a wide variety of neoplasms, including AML and MDS. Inhibition of farnesylation of other proteins besides Ras may account for tipifarnib activity in cells with wild-type Ras.^43^ A number of cell lines are sensitive to farnesyltransferase inhibitors. In a Phase II study of oral tipifarnib, administered to 27 evaluable patients with MDS at a dose of 600 mg twice daily for 4 weeks, every 6-weeks, three patients (11%) responded, including 2 CR and 1 PR. Most common adverse events included myelosuppression, rash, fatigue, and gastrointestinal upset.^53^ A multicenter Phase II study enrolled 82 patients with higher-risk MDS, who received tipifarnib 300 mg orally twice daily for 21 days of each 28-day cycle. Using IWG criteria, there were 12 (15%) CR, and 14 (17%) hematologic improvements.^54^

## Conclusions:

New agents affecting crucial pathogenetic pathways have shown to induce significant clinical responses in MDS and have entered clinical practice, changing the therapeutic paradigm and guidelines. Although some agents have been shown to increase survival, so far none can be considered curative, since responses are usually time-limited and are lost following drug discontinuation. Further developments are definitively required, including combination studies with different active drugs. The use of these new drugs in the setting of allogenic stem cell transplantation is under investigation in selected younger patients. A better understanding of MDS biology will warrant more targeted therapy approaches in the future.

## Figures and Tables

**Table 1:** Main trials using hypomethylating agents in MDS. Diagnosis are listed according to the F.A.B. Classification. CR also include marrow CR, according to modified IWG criteria (2006).

**Trial**	**Patients (n)**	**Diagnosis (n)**	**Treatment**	**CR (%)**	**PR (%)**	**HI (%)**	**Ref.**
CALGB 8421	48	RAEB (23) RAEB-t (24) AML (1)	AZA-SD i.v.	15	2	27	26
CALGB 8921	70	RA/RARS (11) RAEB (19) RAEB-t (16) CMML (14) AML (10)	AZA-SD s.c.	17	0	23	26
CALGB 9221	150* 41	RA/RARS (49) RAEB (70) RAEB-t (33) CMML (20) AML (19)	Aza-SD BSC	9 0	3 0	33 17	24
AZA-001 phase III**	179 179	RAEB (207) RAEB-t (123) CMML (11) AML (2)	AZA-SD Convent. Care	17 8	12 4	49 29	27
GMDSSG/EORTC 06011 °	233	IPSS Int-2 (128) IPSS high (105)	DAC °° BSC	13 0	6 0	15 2	28
MD Anderson	64 14 17	IPSS Int-1 (19) IPSS Int-2 (26) IPSS High (11) Not-appl. (39)	DAC 20 mg/m^2^ iv 5 d 20 mg/m^2^ sc 5 d 10 mg/m^2^ iv 10 d	58	1	14	30
ADOPT	99	RA/RARS (37) RAEB/RAEB-t (51) CMML (11)	DAC 20 mg/m^2^ iv 5 d	32	0	18	31

Aza-SD (standard dose): azacitidine 75 mg/m^2^ daily, subcutaneously for 7 days every 28 days; BSC: best supportive care; convent. care: best supportive care, low-dose cytarabine, or intensive chemotherapy. DAC: Decitabine,

°° 5 mg/m^2^ i.v. over 4 hours, every 8 hours for three days, every 6 weeks, for a maximum of 8 cycles.

* 51 patients received Aza-SD at disease progression, after observation.

** Overall survival and time to AML transformation were significantly longer for the Aza-arm (21.5 versus 11.5 months, p= 0.0045, and 15 versus 10.1 months, p<0.0001, respectively).

° PFS was significanlt longer in the DAC-arm (0.55 versus 0.25 years, p=0.004), while time to AML or death were not significantly different in the two arms.
